# Physical Effects of Radiation Modification of Biodegradable Xylitol-Based Materials Synthesized Using a Combination of Different Monomers

**DOI:** 10.3390/polym13071041

**Published:** 2021-03-26

**Authors:** Marta Piątek-Hnat, Kuba Bomba, Janusz P. Kowalski-Stankiewicz, Jakub Pęksiński, Agnieszka Kozłowska, Jacek G. Sośnicki, Tomasz J. Idzik, Beata Schmidt, Krzysztof Kowalczyk, Marta Walo, Agnieszka Kochmańska

**Affiliations:** 1 Faculty of Chemical Technology and Engineering, West Pomeranian University of Technology, 71-065 Szczecin, Poland; bk34688@zut.edu.pl (K.B.); agak@zut.edu.pl (A.K.); 2 Department of Computer Sciences in Medicine & Education Quality Evaluation, Pomeranian Medical University in Szczecin, 70-204 Szczecin, Poland; janus@pum.edu.pl; 3 Faculty of Electrical Engineering, West Pomeranian University of Technology, 71-313 Szczecin, Poland; jakub.peksinski@zut.edu.pl; 4 Department of Organic and Physical Chemistry, Faculty of Chemical Technology and Engineering, West Pomeranian University of Technology, 71-065 Szczecin, Poland; jacek.sosnicki@zut.edu.pl (J.G.S.); tomasz.idzik@zut.edu.pl (T.J.I.); 5 Department of Chemical Organic Technology and Polymeric Materials, Faculty of Chemical Technology and Engineering, West Pomeranian University of Technology, 71-065 Szczecin, Poland; Beata.Schmidt@zut.edu.pl (B.S.); Krzysztof.Kowalczyk@zut.edu.pl (K.K.); 6 Laboratory for Measurements of Technological Doses, Institute of Nuclear Chemistry and Technology, 03-195 Warszawa, Poland; M.Walo@ichtj.waw.pl; 7 Department of Materials Technology, West Pomeranian University of Technology, 70-310 Szczecin, Poland; akochmanska@zut.edu.pl

**Keywords:** radiation modification, e-beam, xylitol, biodegradable elastomers, mechanical and thermal properties

## Abstract

There is a possibility of obtaining xylitol-based elastomers sharing common characteristics of biodegradability, thermal stability, and elastomeric behavior by using monomers with different chain-lengths. Therefore, we have synthesized eight elastomers using a combination of four different diols (ethanediol, 1.3-propanediol, 1.4-buanediol, and 1.5-pentanediol) and two different dicarboxylic acids (succinic acid and adipic acid). The obtained materials were further modified by performing e-beam treatment with a dose of 100 kGy. Materials both before and after radiation modification were tested by DSC, DMTA, TGA, tensile tests, gel fraction determination, hydrolytic and enzymatic degradation tests, ^1^H NMR and ^13^C NMR and FTIR.

## 1. Introduction

Recent trends in commodity elastomers have focused on obtaining biodegradable materials and, at least partially, monomers from renewable sources. An example of one of these monomers is sugar alcohols.

Elastomers based on sugar alcohols can be divided into two main groups: (1) poly (polyol dicarboxylates) [1,2,3], with the most notable examples being poly(glycerol sebacate) (PGS) [4,5,6,7,8] and poly (xylitol sebacate) [9,10,11,12]; and (2) poly (polyol dicarboxylates-co-diol-dicarboxylates) [13,14,15,16,17,18,19,20].

An important advantage of sugar-alcohol-based polyesters is the possibility to tailor their properties (such as degradation time, glass transition temperature, elongation, and stress at break) for a specific application while retaining their core characteristics of biodegradability, thermal stability, and elastomeric behavior. A number of approaches can be utilized to do so. There are five options regarding poly (polyol dicarboxylates): (1) carrying out the synthesis using the same dicarboxylic acids but different polyols [1,2]; (2) performing the synthesis using the same polyol but different dicarboxylic acids [3,21]; (3) conducting the synthesis using the same monomers but with different stoichiometric ratios [1,12]; (4) copolymerization of those homopolymers [22]; and (5) finally performing the syntheses with different reaction temperatures [4].

In the case of poly (polyol dicarboxylate-co-diol-dicarboxylates), fine-tuning the characteristics can be done by changing the polyol used in the synthesis [13,15,16], utilizing different dicarboxylic acids [18] or different diols [14], and by changing the polycondensation duration [19]. The range of attainable properties widens even further when taking into consideration the possibility of modifying those elastomers with different doses of e-beam radiation [15,16].

Research into e-beam radiation modification of biodegradable polyesters is fairly limited, with poly (lactic acid) [23,24,25] and polycaprolactone [26,27] being the most intensely investigated materials, and other research including poly(butylene succinate) [28], bacterial polyesters-poly (hydroxyalkanoate) [29], and poly-(R)-3-hydroxybutyrate [30].

Regarding the lack of scientific data on radiation modification of biodegradable polyesters, continued research in this field is important. Due to the fact that sugar-alcohol-based elastomers are well-suited for such modification, we decided to study a range of those materials, most of which, to the best of our knowledge, have never been synthesized and radiation modified before. We obtained eight elastomers based on xylitol, two different dicarboxylic acids, and four different diols. Compared to our previous papers [15,16,17,18,19,20], the synthesis method was modified as compared to our previous papers [15,16,17,18,19,20], allowing us to apply a crosslinking time from 12 days to 48 h on average. Moreover, a 100 kGy dose of radiation was chosen based on our previous research [15,16].

## 2. Materials and Methods

### 2.1. Synthesis of Elastomers

All chemicals were purchased from Sigma-Aldrich (St. Louis, MO, USA). In this study, eight poly (xylitol dicarboxylate-diol dicarboxylate) elastomers were synthesized.

Poly (xylitol succinate-co ethylene succinate) (PXESu) was synthesized using xylitol, succinic acid, and ethanediol. Poly (xylitol succinate-co-propylene succinate) (PXPSu) was synthesized using xylitol, succinic acid, and 1.3-propanediol. Poly (xylitol succinate-co-butylene succinate) (PXBSu) was synthesized using xylitol, succinic acid, and 1.4-butanediol. Poly (xylitol succinate-co-pentylene succinate) (PXPeSu) was synthesized using xylitol, succinic acid, and 1.5-pentanediol.

Poly (xylitol adipate-co-ethylene adipate) (PXEA) was synthesized using xylitol, adipic acid, and ethanodiol. Poly (xylitol adipate-co-propylene adipate) (PXPA) was synthesized using xylitol, adipic acid, and 1.3-propanediol. Poly (xylitol adipate-co-butylene adipate) (PXBA) was synthesized using xylitol, adipic acid, and 1.4-butanediol. Poly (xylitol adipate-co-pentylene adipate) (PXPeA) was synthesized using xylitol, adipic acid, and 1.5-pentanediol.

The synthesis process consists of three steps: (1) 9 h esterification reaction in 150 °C in nitrogen atmosphere in a vacuum evaporator; (2) 3 h polycondensation reaction in 150 °C in low-pressure-atmosphere in a vacuum evaporator; and (3) crosslinking reaction of materials cast into silicone forms at 150 °C in a low-pressure-atmosphere in a vacuum dryer.

Samples obtained after polycondensation are called prepolymers which are then cured and crosslinked elastomers are obtained. Then, crosslinked elastomers are modified with e-beam.

The molar ratio of dicarboxylic acid:xylitol:diol was 2:1:1. No catalyst was used for the synthesis. The value of the vacuum applied was 100 mBar during both polycondensation and crosslinking.

### 2.2. Irradiation

After crosslinking, materials were e-beam irradiated in the Institute of Nuclear Chemistry and Technology (Warsaw). A linear electron accelerator Elektronika 10/10 (NPO Torij, Russia) was used to generate a 10 MeV at a 100 kGy dose. Radiation was split into 25 kGy doses. The average set current was 360 mA, and samples were moved with 0.368 m/min speed. 

## 3. Experimental Methods

### 3.1. Nuclear Magnetic Resonance Spectroscopy (NMR)

^1^H and ^13^C NMR spectroscopic measurements were performed on a Bruker DPX 400 AVANCE III HD spectrometer (Bruker, Rheinstetten, Germany), operating at 400.1 and 100.6 MHz, respectively. Approximately 50 mg of each sample was dissolved in 0.7 mL of deuterated chloroform (CDCl_3_). TMS was used as an internal reference, and spectra were acquired in 5 mm probes. For NMR analyses, the MestReNova program (version 12.0.3, Mestrelab, Santiago de Compostela, Spain) was used.

### 3.2. Fourier Transform Infrared Spectroscopy (FTIR)

Analyses of the chemical structures of the polymers were conducted with Fourier transform infrared spectroscopy (FTIR). An Alpha Spectrometer Bruker (Bruker, Germany) was used. Recorded transmission spectra were in the range between 4000 cm^−1^ and 400 cm^−1^ with a resolution of 2 cm^−1^. In order to develop the results, the Omnic 7.3 software by the Thermo Electron Corporation (Waltham, MA, USA) was used. Analyses were performed on the elastomers before and after irradiation.

### 3.3. Differential Scanning Calorimetry (DSC)

In order to determine the thermal properties of the materials, differential scanning calorimetry (DSC) was utilized. TA Instruments apparatus Q2500 (New Castle, DE, USA) was used. Parameters of the analysis were −100 to 100 °C heating cycle and 10 °C/min heating rate. Tests were performed in a nitrogen atmosphere. In order to develop the results, TA Instruments Universal Analysis 2000, 3.9a software (New Castle, DE, USA) was used. Tests were performed on elastomers before and after irradiation.

### 3.4. Dynamic Thermomechanical Analysis (DMTA)

In order to perform dynamic thermomechanical analysis, a DMA Q800 (TA Instruments, New Castle, DE, USA) was used. The temperature range was −100 to 100 °C with 1 Hz frequency. The heating rate was 2 °C/min. Analysis was carried out on nonmodified samples, and samples modified using 100 kGy. The TA Instruments Universal Analysis 2000, 3.9a software used to develop the test results.

### 3.5. Mechanical Properties

Testing of the mechanical properties was performed using Instron 36 instrument (Norwood, MA, USA). Parameters of the tests were 25 °C, 50% of relative humidity, 100 mm/min crosshead speed, and 500 N load cell. Tests were performed in keeping with PN-EN-ISO 526/1:1996 standard. Tests were performed on elastomers before and after irradiation.

### 3.6. Gel Fraction

Each crosslinked polymer sample (about 0.5g) was placed in a container and immersed in tetrahydrofuran for 5 days. Samples were then dried in a desiccator at lowered pressure for 14 days at 25 °C. The following formula was used to calculate the gel fraction content:

(1) $$X\;=\;\frac{m_{0}}{m_{1}}\;\times \;100\%$$

where m_1_-mass of the sample after extraction and m_0_-mass of the sample before extraction

### 3.7. Hydrolytic Degradation

In order to perform the hydrolytic degradation, 10 mm polymer discs were prepared. Samples were placed in a 48-well plate and sterilized with UV light for 20 min in a laminar chamber. After sterilization, each sample was covered with 1.5 mL of phosphate-buffered saline (PBS) (Sigma Aldrich, St. Louis, MO, USA) solution with a pH range of 7.1 to 7.2. The samples were kept at 37 °C for 21 days. Every two days, the samples were sterilized again, and the PBS solution was changed. The following formula was used to calculate mass loss after 21 days:

D = (m_0_ − m_1_)/m_0_ × 100% (2)

where m_0_ is the mass of the sample before degradation, m_1_ is the mass of the sample after degradation, and D is the mass loss.

### 3.8. Enzymatic Degradation

In order to perform the enzymatic degradation, 10 mm polymer discs were prepared. Samples were placed in a 48-well plate and sterilized with UV light for 20 min in a laminar chamber. After sterilization, each sample was covered with 1.5 mL of a solution of porcine lipase in PBS (Sigma Aldrich, Poznań, Poland) solution with pH range of 7.1 to 7.2. The samples were kept at 37 °C for 21 days. Every two days, the samples were sterilized again, and the lipase solution was changed. The following formula was used to calculate mass loss after 21 days:

D = (m_0_ − m_1_)/m_0_ × 100% (3)

where m_0_ is the mass of the sample before degradation, m_1_ is the mass of the sample after degradation, and D is the mass loss.

### 3.9. Thermogravimetric Analysis (TGA)

TGA analysis was performed to analyze the thermal stability of the elastomers. A Q500 TGA instrument (TA Instruments, New Castle, DE, USA), equipped with platinum crucibles, was used. A heating rate of 10 °C was utilized. The temperature range was 25 °C to 600 °C. The weight of the samples was ~15 mg. The test was conducted in dry air atmosphere.

Analyses were performed for nonirradiated, crosslinked elastomer samples which were taken directly after synthesis.

### 3.10. Gel Permeation Chromatography (GPC)

Determination of the molecular weights of the PGBS, PEBS, PXBS, PSBS, and PMBS prepolymers was conducted using gel permeation chromatography (GPC). Styragel column (Waters, Milford, MA, USA) was utilized. Samples were dissolved (1 mg/mL) in tetrahydrofuran (THF).

### 3.11. Scanning Electron Microscopy (SEM)

Field emission scanning electron microscopy (FE-SEM) analysis was carried out using the Hitachi SU-70 microscope (Tokyo, Japan). The samples were coated with palladium-gold alloy thin film using thermal evaporation the physical vapor deposition (PVD) method to provide electric conductivity using JEOL JEE 4x (Tokyo, Japan). SEM analyses were performed at an accelerating voltage of 1 kV, and secondary electron images were acquired.

## 4. Results and Discussion

**Figure 1:**
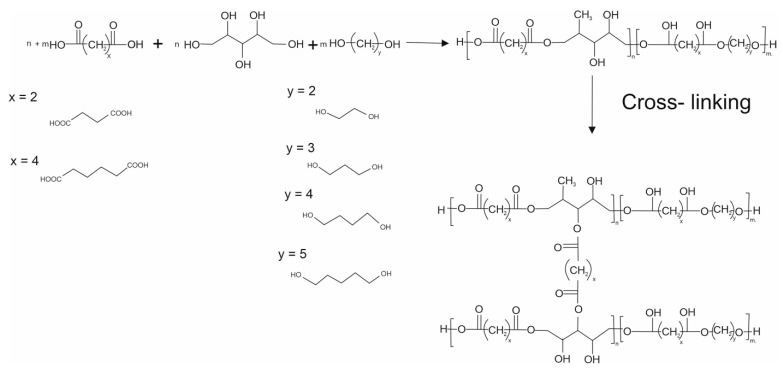
Scheme of the poly (xylitol dicarboxylate-co-diol dicarboxylate) synthesis.

The composition and properties of the elastomers are summarized in Table 1, and a synthesis scheme is shown in Figure 1.

### 4.1. Nuclear Magnetic Resonance Spectroscopy (NMR)

**Figure 2:**
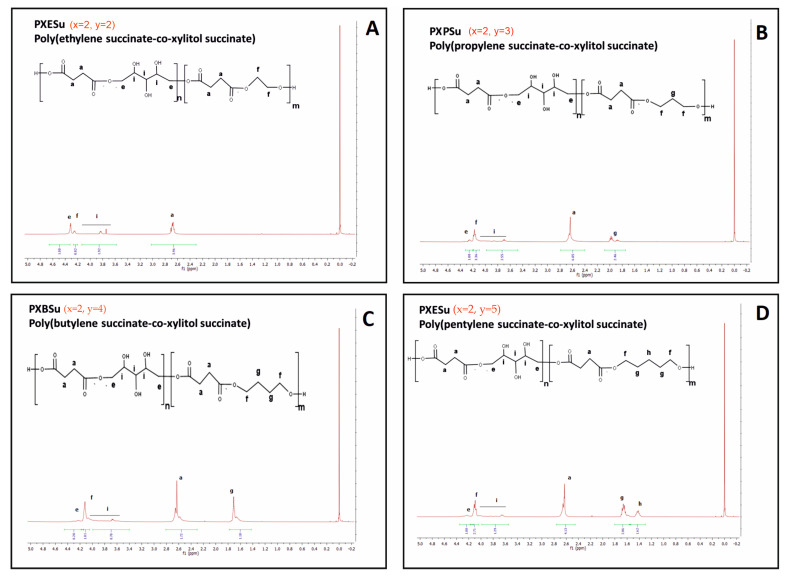
1H NMR of PXESu (**A**), PXPSu (**B**), PXBSu (**C**), and PXPeSu (**D**) prepolymers.

**Figure 3:**
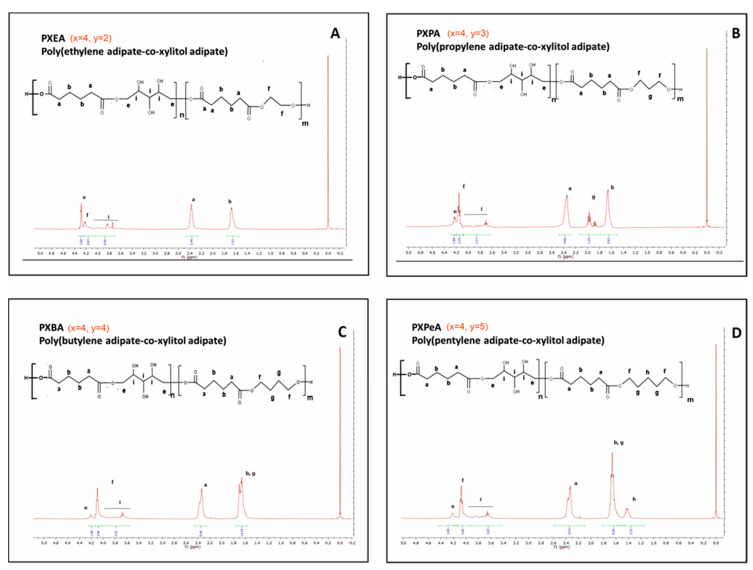
1H NMR of PXEA (**A**), PXPA (**B**), PXBA (**C**), and PXPeA (**D**) prepolymers.

**Figure 4:**
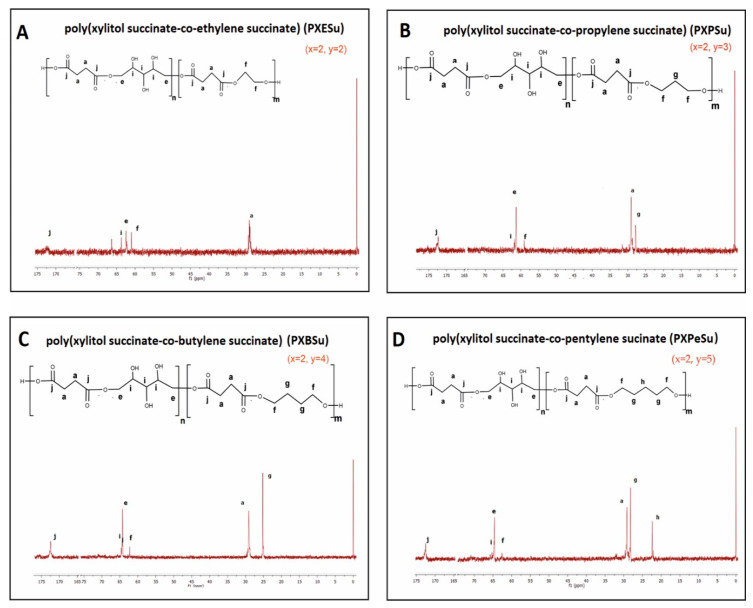
^13^C NMR of PXESu (**A**), PXPSu (**B**), PXBSu (**C**), and PXPeSu (**D**).

**Figure 5:**
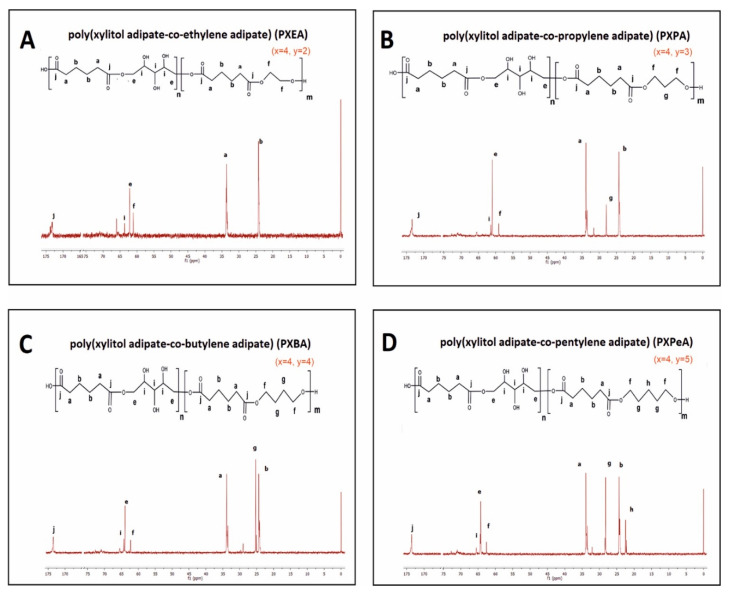
^13^C NMR of PXEA (**A**), PXPA (**B**), PXBA (**C**), and PXPeA (**D**) prepolymers.

To determine the chemical structure of the obtained magnetic nuclear resonance spectroscopy, ^1^H NMR and ^13^C was carried out. The results are presented in Figure 2, Figure 3, Figure 4 and Figure 5.

In ^1^H NMR, peaks ranging from about 2.6 ppm to 1.2 ppm were attributed to −CH2 groups in both diols and dicarboxylic acid peaks at about 1.4 ppm. This association was due to the CH2 (h) group in diol with a peak at about 1.6–1.8 ppm, linked to the CH2 (b) group in dicarboxylic acid and CH2 (g) group in diol. The peak at about 2.4–2.6 ppm was associated with the CH2 (a) group in the dicarboxylic acids. Peaks in the range of 3.6–4.6 ppm were associated with secondary −OH groups in xylitol. The peak at about 4.2 ppm was linked to the ester bond between diol and dicarboxylic acid, and the peak at about 4.4 ppm was due to the ester bond between xylitol and dicarboxylic acid.

By dividing the area of the peak at 4.4 ppm by the area of that at 4.2 ppm, the molar composition of obtained prepolymers was calculated and presented in Table 1.

In ^13^C NMR, the following peaks could be assigned to –CH_2_ groups in aliphatic chains: (1) the peak at about 34 ppm was linked to CH_2_ (a) groups in dicarboxylic acid; (2) the peak at about 28 ppm was connected to CH_2_ (g) group in diol; (3) the peak at about 24 ppm was due to the CH_2_ (b) groups in dicarboxylic acid and; (4) the peak at about 22 ppm was associated with the CH_2_ (h) group in diol. The peak at about 65 ppm was linked to CH_2_OH (i) groups in xylitol. The peak at about 64 ppm was connected to the carbon atom next to the ester bond between xylitol and dicarboxylic acid, and that at about 62 ppm was linked to the carbon atom next to the ester bond between diol and dicarboxylic acid. The peak at about 172 ppm was linked to the carbonyl group.

### 4.2. Fourier Transform Infrared Spectroscopy (FTIR)

**Figure 6:**
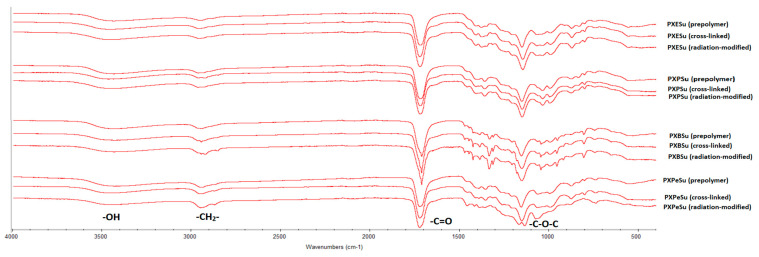
FTIR spectra of PXESu, PXPSu, PXBSu, and PXPeSu prepolymers and polymer crosslinked and polymer after radiation modified.

**Figure 7:**
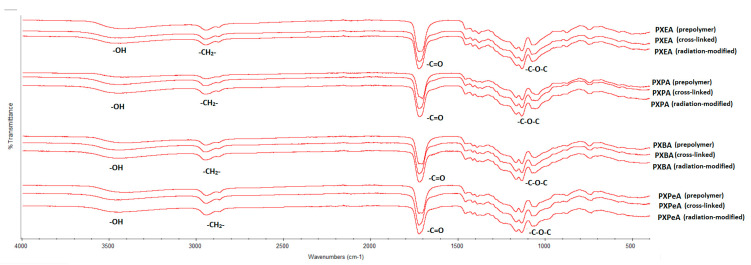
FTIR spectra of PXEA, PXPA, PXBA, and PXPeA prepolymers, polymer crosslinked, and polymer after radiation modified.

Figure 6 and Figure 7 show the FTIR spectra of poly (xylitol dicarboxylate-co-diol dicarboxylate) elastomers. Four signals that are typical for sugar-alcohol-based polyesters can be observed. The signal at 1170 cm^−1^ was linked to −C-O-C groups, that at 1725 cm^−1^ was due to the C = O groups, that at 2930 cm^−1^ was connected to –CH2^−^ groups, and that at 3450 cm^−1^ was linked to nonmolecularly associated –OH groups. The lack of significant changes in the spectra of polymers after radiation-modification showed that the polymers do not degrade as a result of e-beam treatment.

A small decrease in the signal intensities was linked to free −OH groups, and an increase was linked to −C-O-C− groups was seen when comparing the spectra of prepolymers and crosslinked polymers. This occurred due to the creation of crosslinks between polymer chains, which, in turn, resulted from the reaction of hydroxyl groups and left-over unreacted dicarboxylic acid molecules. This change in signal intensities was a confirmation that the crosslinking process had taken place.

### 4.3. Thermal Properties: Differential Scanning Calorimetry (DSC)

**Figure 8:**
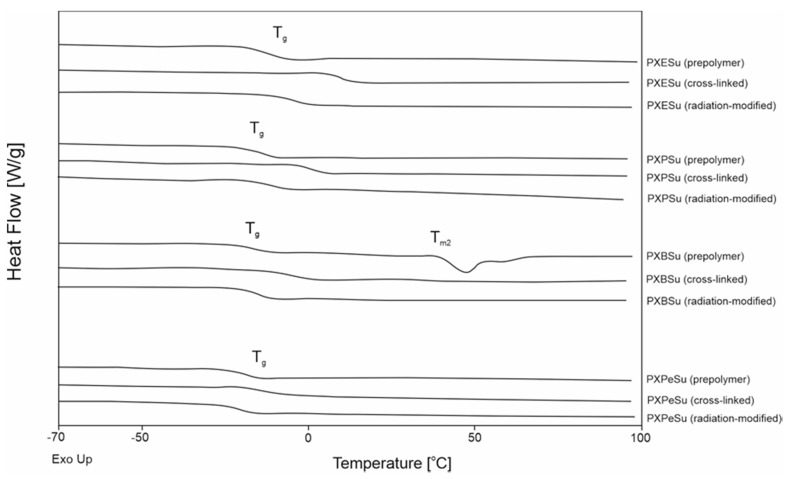
Differential scanning calorimetry (DSC) thermograms for first-heating of PXESu, PXPSu, PXBSu, PXPeSu prepolymers and polymer crosslinked and polymer after radiation modified.

**Figure 9:**
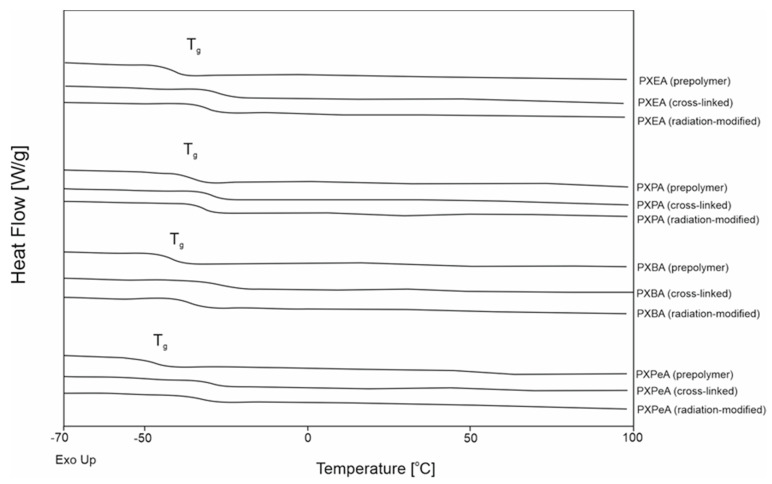
Differential scanning calorimetry (DSC) thermograms for first-heating of PXEA, PXPA, PXBA, and PXPeA prepolymers and polymer crosslinked and polymer after radiation modified.

Differential scanning calorimetry was carried out to determine how the thermal properties of the obtained elastomers were affected by syntheses with different combinations of dicarboxylic acids and diols. The results are presented in Figure 8 and Figure 9 and Table 2.

PXBSu was the only material that exhibited a melting transition. This transition was not present for crosslinked material. All prepolymers, crosslinked polymers, and radiation-modified polymers exhibited glass transition temperatures. Glass transition temperatures exhibited a decrease when both the diol chain length and dicarboxylic acid chain length increased. This was due to the lower mobility of macromolecules when synthesized using longer-chained monomers.

Glass transition temperature also decreased due to the crosslinking process and increased again as a result of radiation-modification.

Changes in heat capacity (∆C_p_) were linked to the grass transition remaining within a similar range for both nonmodified and radiation-modified polyesters. This relationship was due to the lack of change in the content of materials in the amorphous phase. This finding proved that the materials did not degrade as a result of e-beam treatment. This correlates well with the FTIR result, which showed a lack of change in the chemical structure due to radiation-modification, and also proved that the elastomers did not degrade as a result of e-beam treatment.

### 4.4. Dynamic Thermomechanical Analysis (DMTA)

**Figure 10:**
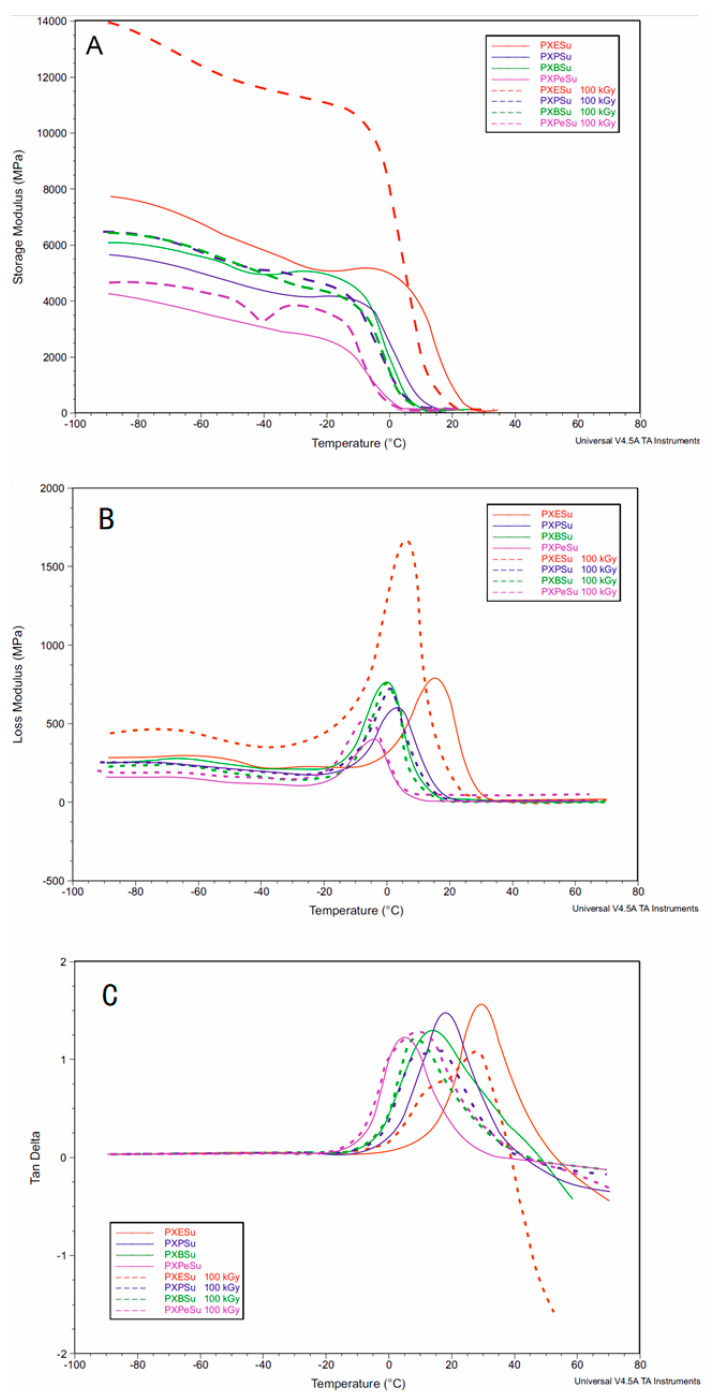
DMTA (**A**) Storage modulus (E′), (**B**) loss modulus (E″), (**C**) loss tangent (tan delta versus temperature) for PXESu, PXPSu, PXBSu, and PXPeSu.

**Figure 11:**
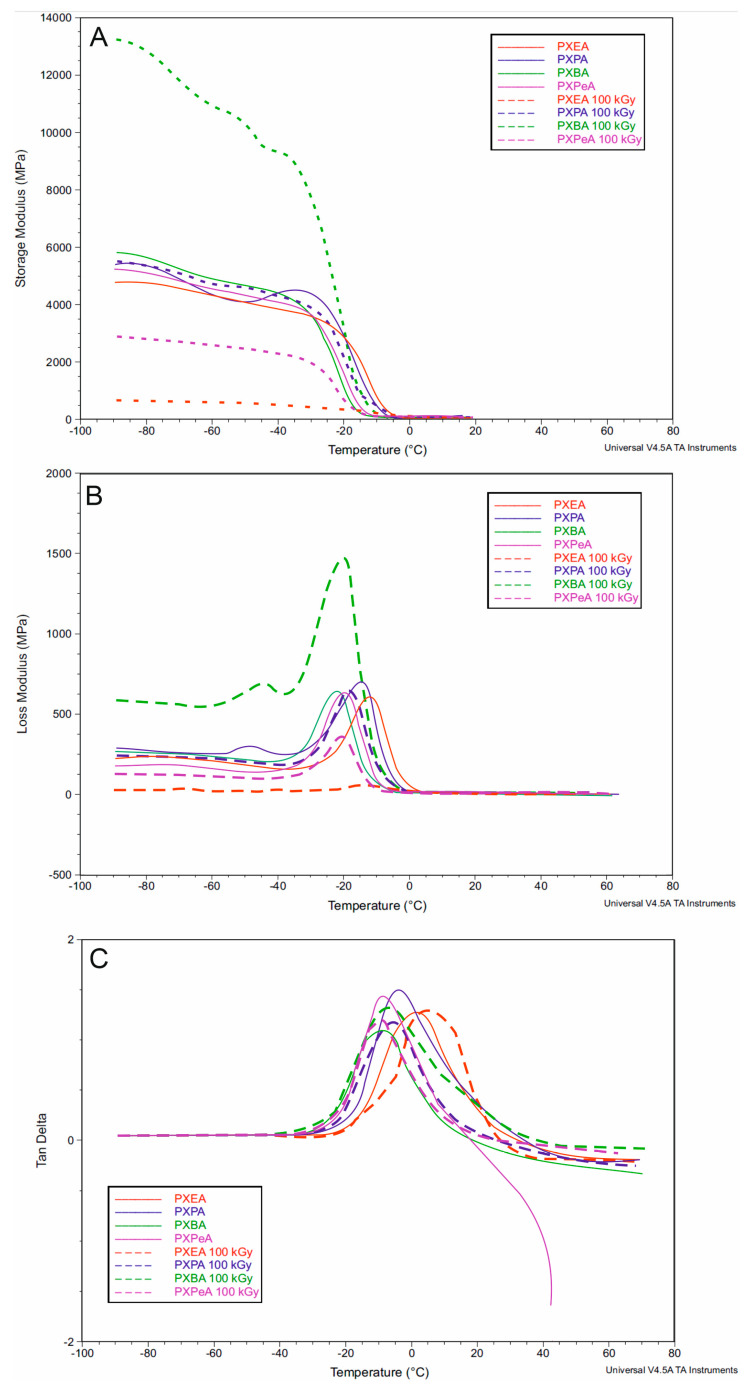
DMTA (**A**) Storage modulus (E′), (**B**) loss modulus (E″), (**C**) loss tangent (tan delta versus temperature) for PXEA, PXPA, PXBA, and PXPeA.

DMTA was used to test the relaxation behavior displayed by PXESu, PXPSu, PXBSu, and PXPeSu (Figure 10) and PXEA, PXPA, PXBA, and PXPeA (Figure 11) before and after radiation modification with 100 kGy. Modulus E′, loss modulus E″, and loss tangent (tan delta) were measured as a temperature function. The materials were in a glassy state in the temperature range between −90 and −20 °C. A viscoelastic relaxation process, associated with the glass transition, was related to a significant decrease of storage modulus which was observed in the temperature range between 20 and 0 °C. The peak maxima of the loss modulus and loss tangent functions associated with α relaxation correspond well with the glass transition temperature determined by DSC. The peak maxima of the loss tangent shift in the direction of lower temperatures was a result of radiation modification. This finding corresponded well with the glass transition temperatures decreasing as a result of radiation modification as confirmed by DSC.

The peak area of the loss modulus and value of storage modulus significantly increased for PXESu and PXBA while decreasing for PXEA materials due to radiation modification. However, radiation-modification did not correlate with a change in mechanical properties, thermal stability, or degradation susceptibility. Overall, all of the DMTA results complemented the results of the DSC analysis.

### 4.5. Mechanical Properties

**Figure 12:**
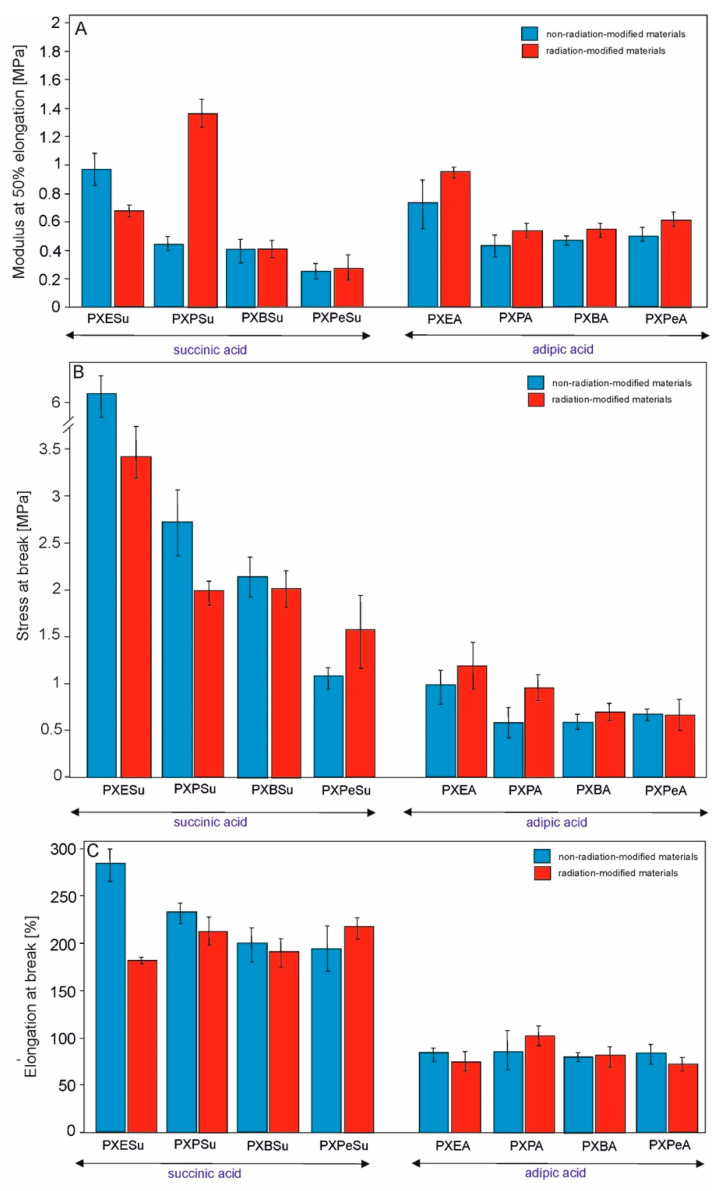
Mechanical properties of PXESu, PXPSu, PXBSu, PXPeSu and PXEA, PXPA, PXBA, and PXPeA nonradiation-modified materials (blue) and radiation-modified materials (red). Tangent modulus at 50% elongation (**A**), stress at break (**B**), and elongation at break (**C**).

Tensile tests determined the influence of monomer chain length on the mechanical properties of the obtained elastomers. The results are presented in Figure 12 and Table 1. The elastomer based on monomers with the shortest chain (PXESu) exhibited the best mechanical properties. Both the elongation at break and stress at break were significantly higher than those of the rest of the materials. Radiation-modification led to an increase of stress at break of PXPeSu, PXEA, PXPA, and PXBA elastomers. The modulus at 50% elongation improved for all materials except for PXESu. PXESu was the only elastomer that did not show any beneficial changes due to the e-beam treatment. Adipic acid-based materials were better suited for radiation modification, but succinic-acid-based elastomers had overall better mechanical properties.

### 4.6. Gel Fraction

**Figure 13:**
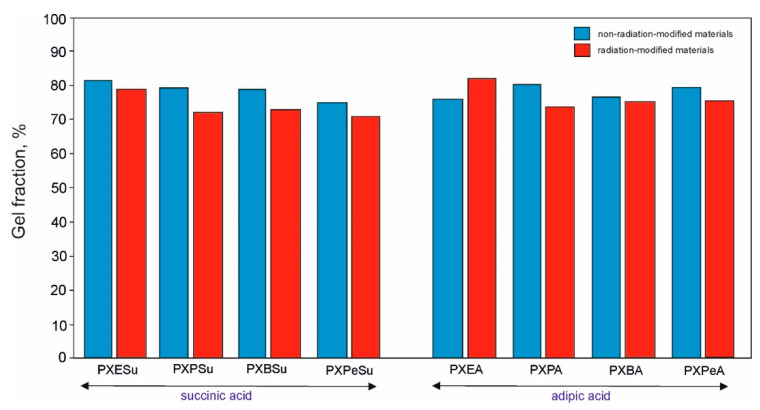
Gel fraction results for PXESu, PXPSu, PXBSu, PXPeSu and PXEA, PXPA, PXBA, and PXPeA nonradiation-modified materials (blue) and radiation-modified materials (red).

The results of gel fraction determination are provided in Figure 13. The gel fraction content of all the materials was within the range of about 71–87%. In all the elastomers except for PXEA, radiation modification led to a decrease of gel fraction content. However, there was no correlation between the value of gel fraction content and its change due to e-beam treatment and the mechanical properties of the elastomers. There was, however, a connection between the decrease of gel fraction content as a result of e-beam treatment and a better susceptibility of the materials to biodegradation.

### 4.7. Biodegradation

**Figure 14:**
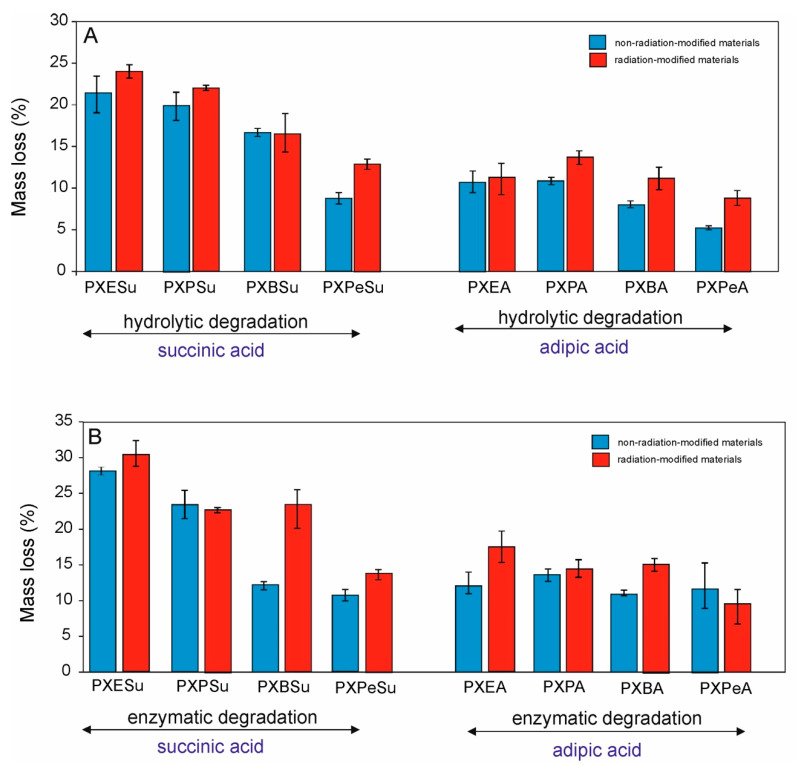
Hydrolytic (**A**) and enzymatic degradation (**B**) of PXESu, PXPSu, PXBSu, PXPeSu and PXEA, PXPA, PXBA, and PXPeA nonradiation-modified materials (blue) and radiation-modified materials (red).

To determine whether the obtained elastomers were biodegradable, and to see how the monomer chain-length affected their susceptibility to degradation, materials were subjected to hydrolytic and enzymatic degradation. Tests were conducted on crosslinked elastomers before and after radiation-modification. The results are provided in Figure 14.

UPTOHERE For hydrolytic degradation, the mass loss after 21 days decreased with the increase of monomer chain length. Mass loss due to the enzymatic degradation, however, showed no correlation with the chain length of the monomers used for the synthesis. It was highest for PXESu and PXPSu and stayed within a similar range for the rest of the materials. The radiation-modification increased the degradation susceptibility of the elastomers. Mass loss due to the enzymatic degradation is higher than in case of the hydrolytic degradation.

### 4.8. Thermogravimetric Analysis (TGA)

**Figure 15:**
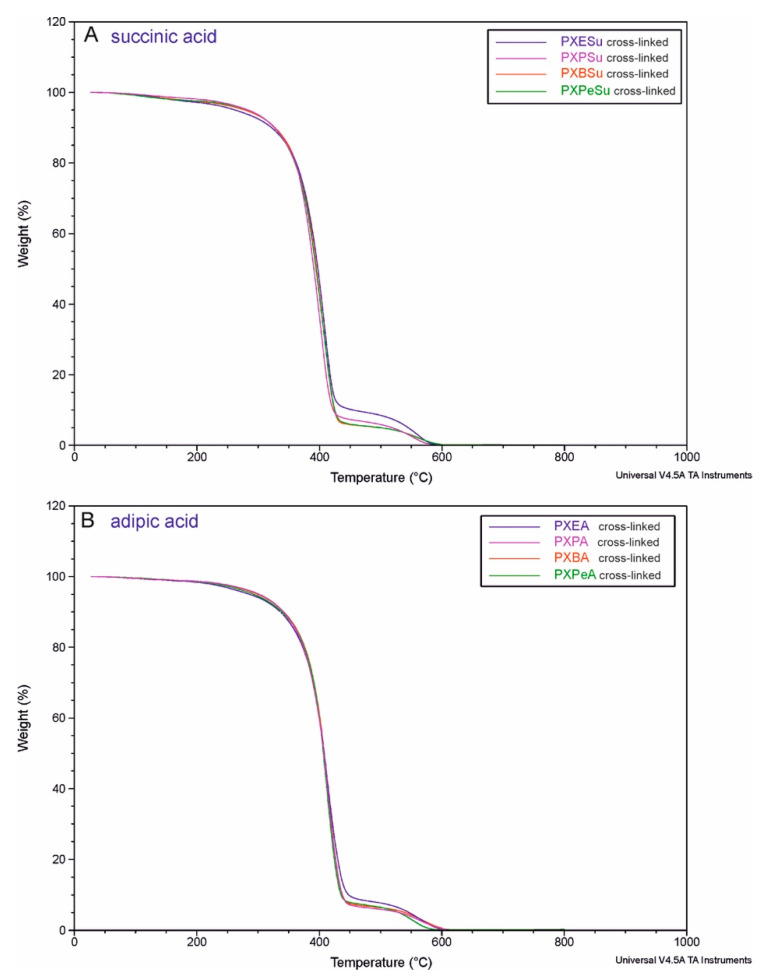
Thermogravimetric analysis (TGA) of PXESu, PXPSu, PXBSu, PXPeSu (**A**) and PXEA, PXPA, PXBA, and PXPeA (**B**) after synthesis.

To determine the thermal stability of crosslinked elastomers, TGA tests were performed. The results are shown in Figure 15. All materials showed similar slopes of the function curve and had thermal stability up to 250 °C, which is significantly higher than both the synthesis and crosslinking temperature and the foreseeable temperature of use.

### 4.9. Scanning Electron Microscopy (SEM)

**Figure 16:**
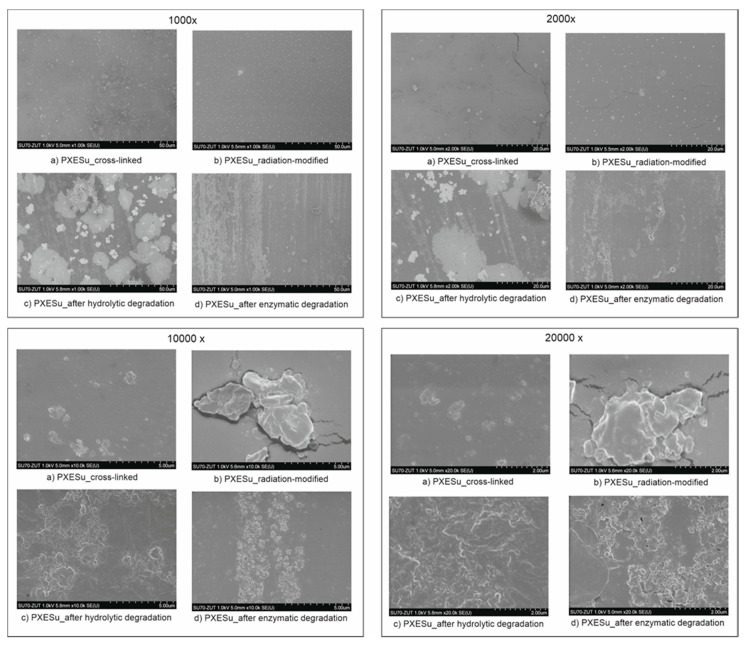
Scanning electron microscopy (SEM) images of PXESu crosslinked (**a**); PXESu radiation-modified (**b**); PXESu after hydrolytic degradation (**c**); PXESu after enzymatic degradation (**d**).

SEM images of polymers before radiation-modification, after radiation-modification, after radiation-modification following hydrolytic degradation, and after radiation-modification following enzymatic degradation were taken to confirm that the physical changes that appeared on the material surface were indeed due to the irradiation process. They are presented in Figure 16.

Physical changes on the material surfaces were present for both PXESu and PXEA. In the case of PXESu, small cracks were visible on the surface, which may have indicated that degradation was taking place. This was mirrored by the reduction of the tensile strength of the material, as determined by tensile tests. Such surface cracks were not present on the surface of PXEA, and this material was characterized improved tensile strength.

Surface hydrolytic and enzymatic degradation was characterized by the presence of salts precipitated from PBS solution and cracking on the surface. This confirmed that the degradation process had taken place in both cases.

## 5. Conclusions

The authors aimed to evaluate the utilization of chain-length monomers to tailor the physicochemical properties of xylitol-based elastomers. With this aim, eight different materials were prepared, and their e-beam treatment was carried out. Their chemical structure was confirmed by both ^1^H NMR and ^13^C NMR. FTIR was used to confirm crosslinking and determined that the polymers did not degrade after radiation-modification. The thermal properties of the were tested by DSC, DMTA, and TGA analyses. The DSC test confirmed the amorphous character of the materials, and the results were well complimented by the DMTA analysis. TGA tests confirmed a good thermal stability of the obtained elastomers. Mechanical test results have shown that adipic acid-based materials are better suited for radiation modification, but succinic-acid-based elastomers have overall better mechanical properties. Elastomers were confirmed to be biodegradable, and their susceptibility to hydrolytic degradation increased as the monomer chain length increased. This relationship was further enhanced by radiation modification. Overall, by choosing appropriate monomers, it is possible to obtain elastomers which are similar but with fine-tuned properties for a specific use. Radiation modification further enhances their properties.

## Figures and Tables

**Table 1:** Composition and selected properties of poly (xylitol dicarboxylate-co-diol dicarboxylate) before and after irradiation.

Material	MC	Stress at Break σ_r_ [MPa]	Elongation at Break ε [%]	Modulus at 50% Elongation E_50% [MPa]	M_w_ (g/mol)	PDI
PXESu (x = 2, y = 2)	1.21	6.60 +/− 0.88	283.2 +/− 17.23	0.97 +/− 0.20	34,000	2.5
PXESu (radiation-modified)	-	3.49 +/− 0.29	182.3 +/− 3.4	0.67 +/− 0.08		
PXPSu (x = 2, y = 3)	0.28	2.72 +/− 0.36	233.7 +/− 11.01	0.44 +/− 0.05	20,000	1.6
PXPSu (radiation-modified)	-	1.99 +/− 0.13	213 +/− 14.4	1.37 +/− 0.5		
PXBSu (x = 2, y = 4)	0.28	2.15 +/− 0.22	200.30 +/− 17.85	0.41 +/− 0.09	21,000	1.5
PXBSu (radiation-modified)	-	2.02 +/− 0.19	192 +/− 14.7	0.4 +/− 0.06		
PXPeSu (x = 2, y = 5)	0.37	1.08 +/− 0.12	196.88 +/− 23.68	0.25 +/− 0.05	24,000	2.1
PXPeSu (radiation-modified)	-	1.57 +/− 0.39	218 +/− 11.25	0.278 +/− 0.09		
PXEA (x = 4, y = 2)	1.21	0.98 +/− 0.18	84.41 +/− 7.05	0.72 +/− 0.18	36,000	2.4
PXEA (radiation-modified)	-	1.19 +/− 0.255	76.7 +/− 10.25	0.95 +/− 0.035		
PXPA (x = 4, y = 3)	0.4	0.59 +/− 0.16	85.32 +/− 20.56	0.44 +/− 0.08	27,000	1.9
PXPA (radiation-modified)	-	0.96 +/− 0.14	103 +/− 10.7	0.54 +/− 0.05		
PXBA (x = 4, y = 4)	0.2	0.59 +/− 0.08	82.08 +/− 4.48	0.47 +/− 0.03	22,000	1.4
PXBA (radiation-modified)	-	0.7 +/− 0.09	81.1 +/− 11	0.54 +/− 0.05		
PXPeA (x = 4, y = 5)	0.26	0.67 +/− 0.6	85.74 +/− 10.46	0.50 +/− 0.06	21,000	1.5
PXPeA (radiation-modified)	-	0.67 +/− 0.17	73.21 +/− 7.12	0.61 +/− 0.05		

Where σ_r_: Stress at break; ε: Elongation at break, E_50%: Modulus at 50% elongation, E_100%; Modulus at 100% elongation; MC-Molar composition (ratio of poly(xylitol dicarboxylate) blocks to poly(diol dicarboxylate) blocks) determined by ^1^H NMR for prepolymers, M_w_-weight average molecular weight, and PDI-polydispersity index.

**Table 2:** DSC termograms of poly (xylitol dicarboxylate-co-diol dicarboxylate) before and after irradiation.

Material	Glass Transition Temperature T_g_ [°C]	Change in Heat Capacity ∆C_p_ [J/g °C]	Melting Temperature T_m2_ [°C]	Melting Enthalphy H_m2_ [J/g]	Degradation Onset Temperature T_d_ [°C]
PXESu prepolymer	−10.21	0.63	─	─	
*PXESu crosslinked*	10.22	0.61	─	─	231
**PXESu (radiation-modified)**	−2.11	0.6050			
PXPSu prepolymer	−14.36	0.6701	─	─	
*PXPSu crosslinked*	1.62	0.55	─	─	220.6
**PXPSu (radiation-modified)**	−9.69	0.6326			
PXBSu prepolymer	−15.35	0.56	47.27	12.59	
*PXBSu crosslinked*	−6.07	0.58	─	─	224.5
**PXBSu (radiation-modified)**	−14.92	0.5953			
PXPeSu prepolymer	−18.11	0.60	─	─	
*PXPeSu crosslinked*	−12.63	0.61	─	─	227
**PXPeSu (radiation-modified)**	−20.23	0.6186			
PXEA prepolymer	−33.29	0.62	─	─	
*PXEA crosslinked*	−16.79	0.53	─	─	213.5
**PXEA (radiation-modified)**	−26.67	0.56			
PXPA prepolymer	−34.86	0.61	─	─	
*PXPA crosslinked*	−28.37	0.58	─	─	209.5
**PXPA (radiation-modified)**	−31.21	0.5580			
PXBA prepolymer	−40.52	0.64	─	─	
*PXBA crosslinked*	−25.73	0.4758	─	─	215.8
**PXBA (radiation-modified)**	−34.99	0.5807			
PXPeA prepolymer	−46.33	0.61	─	─	
*PXPeA crosslinked*	−28.63	0.51	─	─	207.9
**PXPeA (radiation-modified)**	−33.31	0.55			

Where ∆C_p_: change of the heat capacity; T_g_: glass transition temperature; T_m1_: melting temperature, ∆H_m1_: melting enthalphy.

## Data Availability

The data presented in this study is available on request from the corresponding author.
